# Regional Anaesthesia Is Associated with Shorter Postanaesthetic Care and Less Pain Than General Anaesthesia after Upper Extremity Surgery

**DOI:** 10.1155/2016/6308371

**Published:** 2016-11-16

**Authors:** Sven Grauman, Jakob Boethius, Joakim Johansson

**Affiliations:** ^1^Department of Anaesthesia and Intensive Care, Östersund Hospital, Östersund, Sweden; ^2^Department of Surgical and Perioperative Sciences, Anesthesiology and Intensive Care, Unit of Research, Education and Development-Östersund, Umeå University, Umeå, Sweden

## Abstract

*Introduction*. For surgery on the upper extremity, the anaesthetist often has a choice between regional anaesthesia (RA) and general anaesthesia (GA). We sought to investigate the possible differences between RA and GA after upper extremity surgery with regard to postoperative patient comfort.* Methods*. This is a retrospective observational study that was performed at an acute care secondary referral centre. One hundred and eighty-seven procedures involving orthopaedic surgery on the upper extremity were included. The different groups (RA and GA) were compared regarding the primary outcome variable, length of stay in Postanaesthesia Unit, and secondary outcome variables, opioid consumption and nausea treatment.* Results*. RA was associated with significantly shorter median length of stay (99 versus 171 minutes). In the GA group, 32% of the patients received opioid analgesics and 21% received antiemetics. In the RA group, none received opioid analgesics and 3% received antiemetics.* Conclusion*. In this observational study, RA was superior to GA for surgery of the upper extremity regarding Postanaesthesia Care Unit length of stay, number of doses of analgesic, and number of doses of antiemetic administered.

## 1. Introduction

Regional anaesthesia (RA) is used more commonly than general anaesthesia (GA) for surgery on the lower extremity [1]. RA is believed to be associated with less postoperative pain, less resource utilisation in the PACU (Postanaesthesia Care Unit), and fewer complications.

There is not a similar preference for RA in surgery of the upper limb [2, 3].

However, for surgery on the upper extremity, RA has gained popularity during recent years, probably depending on increase in ultrasound (US) availability [2–4].

The brachial plexus is the dominant type of RA used for surgery on the upper extremity. There are a number of different methods of brachial plexus block (interscalene, supraclavicular, infraclavicular, and axillary) and also different methods to ensure deposition of local anaesthetics in proximity to the nerves (anatomical landmarks, nerve stimulator, and/or US). It is possible that this heterogeneity of techniques has hampered the development of a general recommendation on anaesthetic preference for surgery of the upper extremity [1, 5].

RA is an attractive alternative to GA since it can be associated with less perioperative resource utilisation [1, 5]. The risk of perioperative complications is generally considered to be lower with RA as compared to GA [1, 5–7].

It has been shown that RA decreases postoperative pain and time spent in the PACU after hand and shoulder surgery [8–11]. There is less consensus regarding the rate of complications associated with different brachial plexus approaches [2–4, 7]. It is likely that such information, even if it is found, is outdated due to the fast development of the US-guided technique [2–4].

Most studies comparing RA with GA for upper limb surgery are conducted in specialised clinics. Thereby, it is uncertain if the results may be applied to larger, nonspecific units with a bulk of other cases, a mix of urgent and elective procedures, and with a proportion of the physicians in training.

Despite the recent advancements in techniques for RA, postoperative pain and nausea continue to be a major problem [12]. Resource utilisation (PACU LOS (length of stay) and opioid and antiemetic consumption) may reflect the wellbeing of patients.

We wanted to explore the impact of the choice of anaesthesia (RA versus GA) on resource utilisation in the PACU.

## 2. Methods

Ethical approval for this study (2014/396-31) was provided by the Regional Ethics Review Board (Etikprövningsnämnden Umeå, Samverkanshuset, 901 87 Umeå, Sweden). Due to the observational design of the study and no handling of sensitive data, this study did not require informed consent from participants.

This is a retrospective case control study. Our hospital has 10 theatres with mixed cases from all surgical specialties except neurosurgery and thoracic surgery. A total of 8000 procedures are performed annually. The anaesthetists perform all types of anaesthetics, in addition to intensive care. All the RAs and GAs in this study were done in a setting where all physicians, including residents, rotate between all the different positions. No personnel knew of the study at the time of the anaesthesia. Our PACU is open weekdays from 07.00 to 21.00 and takes care of the patients until they are ready to go home (outpatients) or to the ward (inpatients).

A power calculation based on 100 minutes in the PACU for the RA group and 150 minutes for the GA group, 80 minutes standard deviation, power 80%, and level of significance 0.05 revealed that a sample size of 41 in each group was sufficient.

We included all patients in our department that had orthopaedic surgery done on or distal to the elbow from January 1 to June 30, 2012, that were 21 years or older. We did not include cases operated on during on-call hours. Other exclusion criteria were concomitant surgical procedures not on the upper extremity during the same operation, patients not planned for PACU admission after the surgery, or those in which a combination of RA and GA was planned beforehand. If the same patient had more than one procedure on the upper extremity during the study, all such procedures were registered as separate cases. Patient characteristics and all other relevant information were collected from the medical records database.

Two groups were identified to categorise the patients.

1. General anaesthesia (GA), defined as anaesthesia with the use of an endotracheal tube or a supraglottic laryngeal mask. Induction and maintenance of anaesthesia was performed with propofol or thiopental and sevoflurane. Perioperative pain control was achieved with fentanyl, alfentanil, and/or morphine.
2. Regional anaesthesia (RA), defined as a brachial plexus block with local anaesthetics. Ropivacaine was the drug used in almost all cases. Sedation with midazolam or propofol was given in some of these cases.

Almost all patients in the GA group and none in the RA group received local anaesthetics in the surgical area in conjunction with the above-described strategy.

The outcome variables were

- PACU length of stay,
- number of doses of opiates given in the PACU, defined as an occasion with intravenous administration of morphine, ketobemidone, alfentanil, or fentanyl, regardless of the choice of drug and dose,
- Number of doses of antiemetics given in the PACU, defined as an occasion with intravenous administration of haloperidol, granisetron, ondansetron, betamethasone, or metoclopramide, regardless of the choice of drug and dose,
- Regional anaesthesia performed in the PACU (“rescue RA”), defined as a brachial plexus or other conduction block during the time spent in the PACU,
- Unplanned overnight admission to the hospital postoperatively.

Other collected data were ASA-class, duration of the surgical procedure, age, planned out- or inpatient surgery and type of surgery (fracture or no fracture surgery).

### 2.1. Statistical Analysis

Median and interquartile range (ICR) or mean and confidence interval (CI) were used to describe the parameters. For comparison between groups, we used the Mann–Whitney or *t*-test as appropriate. For comparison of binary variables, Fischer's exact test was used. The eight patients with failed RA (that had a GA in addition) are included in the RA group in order to perform an intention to treat analysis (ITT).

A multiple linear regression was performed on the primary outcome (PACU length of stay), looking at the possibly independent variables RA/GA, age, sex, time of surgery, and fracture/no fracture.

## 3. Results

**Figure 1:**
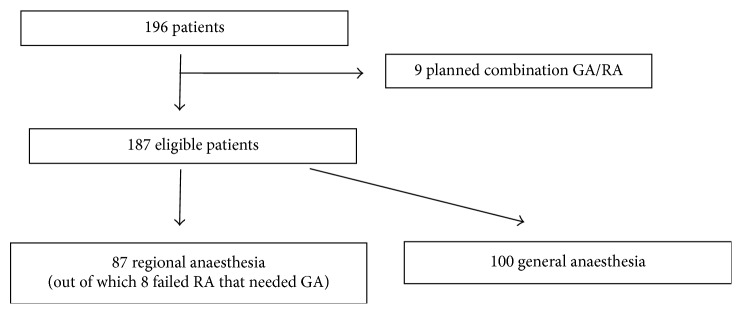
CONSORT-diagram illustrating the study. The 8 patients with failed RA that needed unplanned general anaesthesia was still included in the RA group for an intention to treat analysis.

Figure 1 shows how the study groups were defined. 187 patients were included, 87 in the group RA and 100 in the group GA.

The RA group was significantly older (*p* > 0.001), of a significantly higher ASA-class (*p* = 0.023), and had a longer a duration of surgery (*p* = 0.029) than the GA group, but there was no difference in proportion of planned outpatient surgery or proportion of fracture surgery (Table 1).

The primary outcome variable, PACU length of stay, was significantly shorter after RA than GA (Table 2).

The secondary outcome, administration of opiates and antiemetics, differed significantly between the two groups (Table 2).

Thirteen patients (13%) in the GA group had rescue RA in the PACU and ten (10%) were converted from planned outpatient to inpatient surgery (Table 2).

A multiple regression analysis revealed that independent factors significantly associated with PACU LOS were RA/GA, fracture surgery, and duration of surgery but not age or sex (Table 3).

This multiple regression also suggested looking closer at the group that had surgery in relation to a fracture, since this factor also was related to a longer time spent in the PACU.

A univariate subgroup analysis showed that, in cases with intended RA, the difference in PACU LOS between fracture and nonfracture cases was small (109 versus 94 minutes). The GA group on the other hand doubled their PACU LOS (248 versus 124) (Table 4) if their surgery was in relation to a fracture.

Table 5 demonstrates that in the case of an intended RA other procedures related parameters do not matter that much. On the other hand, for cases where GA was intended, duration of surgery and fracture/nonfracture surgery were both markedly related to PACU LOS.

## 4. Discussion

Our results show that patients that had RA, as compared to those that had only GA, spent a shorter time in the PACU and consumed less analgesic and antiemetic drugs, suggesting less experienced pain and less experienced postoperative nausea and vomiting.

The results corroborate earlier studies to some degree, confirming superiority of an RA approach in upper extremity surgery [1, 5–7]. Despite this knowledge, RA remains underused for different reasons [12]. We theorize that there may be some apprehension to use RA outside of specialised units. What is new is that our study was performed in a secondary referral unit (regional acute care hospital), where surgery on the upper extremity constitutes a small part of the total number of procedures. The procedures involved are both urgent and elective and we have no subspecialised anaesthetists that handle these cases. In addition, approximately 25% of our staff of physicians were residents, representative for a typical acute care general facility. This makes the results applicable to the great majority of care facilities, not only to specialised units.

In the RA group, there was no need for postoperative analgesic intervention and virtually no antiemetic drugs.

From Tables 4 and 5 it is reasonable to conclude that RA intention is most important for fracture surgery, supposedly because of more postoperative pain compared to nonfracture surgery.

There are reasons to believe that the increasing use of US for regional blocks has had an impact on procedure related parameters, such as the learning curve and success rate. All our blocks were done with the support of US and our standard block for arm surgery is the supraclavicular brachial plexus block. The success rate was 92% during this study period, defined as surgery without GA when RA alone was planned on beforehand.

The study has some strengths. It is consecutive and cases included were anaesthetised under real working conditions by nonspecialist regional anaesthetists, including physicians under education. Further, it was not known to participants, anaesthetists, or personnel in the PACU that the case was to be included in a study.

The primary outcome variable, PACU LOS, is somewhat complicated to assess. It is affected by many factors not related to the surgery itself; for example, the surgeon often wants to speak to the patient before he/she leaves the PACU or a postsurgical X-ray often is warranted. In addition to these factors, the outpatients will have to wait for home transportation at the PACU. It is reasonable to suppose that these factors affect both the RA- and the GA group to the same degree and hence that this factor did not have any major effect on the results.

The study also has several weaknesses. Since it is a retrospective study, there was no randomisation; hence the choice between RA and GA was done at the presurgical visit by the anaesthetist. This may introduce confounding variables and the results may not have been the same with randomisation. As can be seen from Table 1, the RA group was significantly older and had longer duration of surgery, so groups were not matched perfect.

As it is a retrospective study, there was no uniform protocol for induction and maintenance of GA and no strict protocol for the blocks.

Furthermore, the study only included patients operated at daytime since our PACU is closed at night. The same personnel join the operation theatre and supply Postanaesthesia Care during on-call hours. It is reasonable to believe that the results would have been the same if on-call hours would have been studied as well.

The outcome variable used for postoperative pain was number of doses of opioids administered. If a patient has severe pain in the PACU, this may call for a single larger dose. Thereby the results may have differed in that patients in severe pain had relatively fewer doses administered. If this was so, it would have counteracted the results found and hence it is not a major limitation.

In light of the exceptional development of RA in recent years, a prospective randomised trial, testing the hypothesis that we have tried to explore here, is indeed warranted and could probably add substantial evidence to the establishment of a gold standard regarding the choice between RA and GA for different types of surgery on the upper extremity.

The results of this study are applicable to a nonspecialised unit where this specific type of surgery constitutes a small part of all procedures and where anaesthetists are not subspecialised, are on a rotating schedule, and include physicians under training.

On this behalf, we conclude that this observational study supports the use of regional anaesthesia as opposed to general anaesthesia for orthopaedic surgery on or below the elbow of the arm. Regional anaesthesia was associated with shorter time spent in the Postanaesthesia care unit and less administered doses of opioids and antiemetics.

## Figures and Tables

**Table 1:** Demographics and study variables in the two groups.

	RA	GA	*p*
*n*	87	100	—
Sex (male, %)	47	45	0.883
Age (years, median, IQR)	60 (54–70)	54 (33–63)	<0.001
ASA (mean, CI)	1.80 (1.66–1.95)	1.59 (1.47–1.71)	0.023
Outpatient (%)	80	77	0.381
Duration of surgery (minutes, median, IQR)	87 (59–117)	74 (42–105)	0.029
Fracture surgery, yes/no	44%	39%	0.309

ASA, American Society of Anesthesiologists.

**Table 2:** Outcome in the two groups.

	RA	GA	*p*
*n*	87	100	—
PACU LOS (minutes, median, IQR)	99 (70–136)	171 (115–251)	<0.001
Opiates in PACU, yes/no	0	32%	<0.001
Opiates, mean number of doses in PACU	0	0.53	na
Antiemetics in PACU, yes/no	3%	21%	<0.001
Antiemetics, number of doses in PACU	0.04	0.44	<0.001
Rescue block in PACU, yes/no	0	13%	<0.001
Unplanned stay overnight	0	10%	0.002

PACU, Postanaesthesia Care Unit. LOS, length of stay. Na, not applicable.

**Table 3:** Multiple regression on PACU-time.

	Coefficient of regression	*p*
Age	−0.06	0.32
Sex	0.01	0.82
Fracture surgery (yes/no)	0.33	<0.001
Duration of surgery	0.15	0.025
RA/GA (yes/no)	0.46	<0.001

RA, regional anaesthesia. GA, general anaesthesia. PACU, Postanaesthesia Care Unit.

**Table 4:** Univariate analyses within the subgroups RA/GA concerning fracture surgery or not.

	RA (*n* = 87)			GA (*n* = 100)		
	Fracture (*n* = 38)	No fracture (*n* = 49)	*p*	Fracture (*n* = 39)	No fracture (*n* = 61)	*p*
PACU LOS (min)	109 (80–149)	94 (61–121)	0.057	248 (186–300)	124 (102–180)	< 0.001

Median (IQR). PACU, Postanaesthesia Care Unit. LOS, length of stay.

**Table 5:** Multiple regressions on PACU length of stay for subgroups RA and GA.

	RA (*n* = 87)		GA (*n* = 100)	
	Coefficient of regression	*p*	Coefficient of regression	*p*
Age	0.09	0.45	−0.09	0.28
Sex	0.13	0.25	−0.07	0.41
Duration of surgery	−0.04	0.73	0.25	0.01
Fracture surgery (yes/no)	0.25	0.03	0.42	<0.001

PACU, Postanaesthesia Care Unit. RA, regional anaesthesia. GA, general anaesthesia.
